# Comparison of the Gut Microbiota in Healthy Infants With Different Delivery Modes and Feeding Types: A Cohort Study

**DOI:** 10.3389/fmicb.2022.868227

**Published:** 2022-05-26

**Authors:** Jingran Ma, Zhenghong Li, Wenjuan Zhang, Chunli Zhang, Yuheng Zhang, Hua Mei, Na Zhuo, Hongyun Wang, Dan Wu

**Affiliations:** ^1^State Key Laboratory of Complex Severe and Rare Diseases, Department of Pediatrics, Peking Union Medical College Hospital, Chinese Academy of Medical Science and Peking Union Medical College, Beijing, China; ^2^Department of Neonatology, Inner Mongolia People's Hospital, Hohhot, China; ^3^Department of Neonatology, The Affiliated Hospital of Inner Mongolia Medical University, Hohhot, China; ^4^Department of Neonatology, Inner Mongolia Maternal and Child Health Hospital, Hohhot, China

**Keywords:** gut microbiota, vaginal delivery, cesarean section delivery, breastfeeding, formula, infants

## Abstract

To compare the gut microbiota of healthy infants based on specific interactions of delivery modes and feeding types, we recruited 62 healthy babies who were followed up for 2 years from our previous cohort study of 91 infants (the rest were lost to follow-up). They were exclusively fed breast milk or specific formulas for more than 4 months after birth. The fecal bacterial composition was tested at 40 days, 3 months, and 6 months of age. Solid foods were introduced from 4 to 6 months of age and thus did not affect the microbiota before 4 months of age. According to the different delivery modes (i.e., vaginal delivery, VD, or cesarean section delivery, CS) and feeding types (i.e., breast-fed, br, or formula-fed, fo), the infants were assigned to four different groups, namely, the VD-br, VD-fo, CS-br, and CS-fo groups. We found that at 40 days of age, the α diversity (reported as the Shannon index) was lower in the br infants than in the fo infants. At 3 months of age, the α diversity was significantly lower in the CS-br group, although significant differences were not observed after solid food introduction. *Bifidobacterium* represented the most predominant genus in all groups at all time points, followed by *Enterobacteriaceae*. At 40 days of age, the abundance of *Bifidobacterium* was much higher in the CS-br group than in the CS-fo group but did not differ between the VD-br and VD-fo groups. The differences in *Bifidobacterium* disappeared at 3 and 6 months of age among the different groups. At 40 days of age, the abundance of *Streptococcus* and *Enterococcus* was much lower in the br infants than in the CS-fo group. At 3 months of age, *Enterococcus* was significantly lower in the CS-br group than in the fo infants, although for infants delivered by VD, the difference between feeding types was not significant. The specific interaction of delivery modes and feeding types has a large impact on the infants' gut microbiota. Breastfeeding and VD may decrease the potential adverse effects of formula feeding or CS delivery on gut microbiota, thus leading to a more stable and beneficial gut environment for infants.

## Introduction

The human fetus is thought to develop within a bacteria-free environment (Dominguez-Bello et al., 2010). After birth, the infant gut is rapidly colonized through a complex process that depends on multiple overlapping factors, including the mode of delivery and type of feeding (Pannaraj et al., 2017). The first year of life is vital to the development and establishment of gut microbiota (Levin et al., 2016; Laursen et al., 2017), which is of low diversity at birth but evolves into a more complex and adult-like composition by 1–2 years of age (Stewart et al., 2018).

The development of gut microbiota in early life influences later health (Derrien et al., 2019), with this microbiota affecting immune system maturation and nutrient absorption and preventing pathogen colonization. Many studies have shown an association between human gut microbiota and chronic noninfectious diseases, including obesity, atopic diseases, and chronic inflammatory diseases. Moreover, a window of opportunity occurs for the regulation of the gut microbiota in early life to promote long-term health (Liu et al., 2016).

The neonate is exposed to a wide array of microbiota upon delivery, and the mode of delivery strongly affects the gut microbiota in neonates. Compared with vaginally delivered infants, the gut microbiota of infants delivered by cesarean section (CS) is significantly less similar to the microbiota of their mothers (Bäckhed et al., 2015). Vaginally delivered infants acquire bacterial communities that resemble the vaginal microbiota of their mothers, and CS-delivered infants harbor bacterial communities similar to those found on the skin surface (Dominguez-Bello et al., 2010).

Human breast milk has superior effects on the infants' health compared with formula, such as protecting the barrier integrity and mucosal defenses of the intestinal tract (Stewart et al., 2018), as well as influencing health-promoting microorganisms by immunoactive factors, such as polymeric IgA (pIgA), antibacterial peptides, and components of the innate immune response (Walker and Iyengar, 2015). While the gradient composition of commercial formulas is increasingly close to that of breast milk, the gut microbiota of breast-fed (br) and formula-fed (fo) babies remain distinct (Baumann-Dudenhoeffer et al., 2018). Studies of gut microbiota in babies exclusively fed breast milk or formula are rare and mostly on a small scale. Our previous study of exclusively br or fo infants presented a more accurate gut microbiota than other research articles (Ma et al., 2020) in which babies were partially br or fo.

Different interactions observed with different feeding types and delivery modes have varying impacts on infant gut microbiota; however, few studies have mentioned these effects. To gain a better understanding of how the specific interaction of feeding patterns and delivery modes affects the gut microbial composition, we conducted a reanalysis to detect the gut microbiota in babies who were exclusively fed human milk or a certain kind of formula for more than 4 months after birth and followed up for 2 years and reported to be healthy without severe infectious or allergic diseases. Moreover, solid foods were introduced from 4 to 6 months of age and thus did not affect the microbiota before 4 months of age, which ruled out the impact of solid foods on microbiota in our study. These babies were enrolled in the North China Regional Union of Neonatologist Research Registry for healthy infants with different feeding types.

## Patients and Methods

We conducted a prospective study detecting gut microbiota in babies with different delivery modes and feeding types of either human milk or formulas exclusively for more than 4 months after birth.

### Study Population

Inclusion criteria were the following: (1) Healthy, full-term, newborn babies who were followed up for 2 years after birth. (2) Birth weight was ≥ 2.5 kg. (3) Babies were born between December 2016 and December 2017 in Peking Union Medical College Hospital, Inner Mongolia People's Hospital, The Affiliated Hospital of Inner Mongolia Medical University, and Inner Mongolia Maternal and Child Health Hospital.

Group assignment. According to different delivery modes (i.e., VD or CS) and whether they were exclusively br or fo for more than 4 months after birth, they were assigned to four different groups as VD-br, VD-fo, CS-br, and CS-fo groups. Babies in the breastfeeding group were fed breast milk exclusively for more than 4 months after birth. They were recruited in their regular follow-up for 40 days of age if they were fed breast milk exclusively at that time. Fo babies were fed with formula due to mother's disease or medicine, and other objective reasons were potential subjects of our study. They were recruited before or right after birth. Parents chose formula A (containing α lactalbumin and β casein) or B (containing α lactalbumin, β casein, as well as 1, 3-olein-2-palmitin) voluntarily after they signed informed consent. Both formulas were market products with no reported adverse events.

Exclusion criteria were the following: (1) gestational age <37 weeks; (2) birth weight was <2.5 kg; (3) babies suffered from a serious disease, such as heart failure, metabolic diseases, and congenital intestinal malformations; (4) babies from the br group could not be fed breast milk exclusively for 4 months for any reason; (5) babies from formula A and B fed groups changed formula before 4 months for any reason; (6) babies who were not followed up for 2 years after birth or had severe or chronic diseases during follow-up.

### Study Design

All infants were evaluated for 40 days, 3 months, and 6 months of age. Clinical data and fecal samples were collected at each time point. Similar solid foods, such as infant cereals, purees, and smashed fleshes, were introduced to infants aged 4–6 months. The type and supplement order of solid foods were following the feeding guide for babies by the Chinese Nutrition Society in 2015.

Clinical data. Clinical data were collected, including mothers' conditions, such as combined diseases, antibiotics usage, age, height, weight, and weight gain during pregnancy; and babies' conditions, including mode of delivery, gestational age, gender, weight, length, head circumference, antibiotics usage, household siblings, pets, district, vitamin D supplementation, defecating frequency, stool property, and infections.

### Sample Collection

Fecal samples were collected from all infants at 40 days, 3 months, and 6 months from birth. All the samples were kept in a sterile container, immediately stored in a refrigerator at −70°C, and sent to Beijing for testing collectively.

### DNA Extraction and Sequencing

Extraction of genome DNA: Total genome DNA from samples was extracted using the CTAB/SDS method. DNA concentration and purity were monitored on 1% agarose gels. According to the concentration, DNA was diluted to 1 ng/μl using sterile water. (2) Amplicon generation: 16S ribosomal RNA (rRNA) genes of the V4 region were amplified using a specific primer (515F-806R) with the barcode. All PCR reactions were carried out with Phusion High-Fidelity PCR Master Mix (NEW ENGLAND BIOLABS). (3) PCR products quantification and qualification: The same volume of 1 × loading buffer (contained SYB green) was mixed with PCR products, and electrophoresis was carried out on 2% agarose gel for detection. Samples with the bright main strip between 400 and 450 bp were chosen for further experiments. (4) PCR products mixing and purification: PCR products were mixed in equidensity ratios. The Qiagen Gel Extraction Kit (QIAGEN, GERMANY) was used to purify ten mixture PCR products. (5) Library preparation and sequencing: Sequencing libraries were generated using the TruSeq DNA PCR-Free Sample Preparation Kit (ILLUMINA, USA) following manufacturer's recommendations, and index codes were added. The library quality was assessed using the Qubit 2.0 Fluorometer (THERMO SCIENTIFIC) and Agilent Bioanalyzer 2100 system. At last, the library was sequenced using an Illumina MiSeq platform.

16S rRNA gene sequence analysis: Paired-end reads were assigned to samples based on their unique barcode and truncated by cutting off the barcode and primer sequence. Quality filtering on the raw tags was performed under specific filtering conditions to obtain the high-quality clean tags (Bokulich et al., 2013) according to the QIIME (version 1.7.0, https://qiime.org/index.html) (Caporaso et al., 2010) quality-controlled process. A total of 20,383,186 reads (median 84,737 reads per sample) were obtained from 16S rRNA gene sequencing. The sequence analysis was performed using the Uparse software version 7.0.1001 (Edgar, 2013). Sequences with ≥ 97% similarity were assigned to the same OTUs. A representative sequence for each OTU was screened for further annotation. For each representative sequence, the SILVA Database (DeSantis et al., 2006) was used based on the RDP classifier version 2.2 (Wang et al., 2007) algorithm to annotate taxonomic information. We compared differences in α diversity using Faith's phylogenetic diversity. The β diversity was evaluated using the principal coordinate analysis (PCoA) and PERMANOVA statistics on unweighted and weighted UniFrac distances.

### Statistical Analysis

All statistical analyses were performed using IBM SPSS version 20.0 (IBM CO., ARMONK, NY, USA). Categorical variables were presented as proportions (percentages), and continuous variables were presented as (means ± standard deviation) or median (interquartile range). Normally distributed variables were statistically tested by a two-tailed *t*-test for two independent groups or a one-way analysis of variance (ANOVA) for multiple independent groups. Nonnormal distributed variables were tested using the Kruskal-Wallis test. Intergroup differences were evaluated using the χ2 test for categorical variables. Correlation analyses were performed using the Kendall test for categorical variables and the Spearman test for continuous variables. A standard *P* ≤ 0.05 was considered significant.

## Results

### Basic Characteristics

Among 64 infants who were followed up at 17–33 months of age, two patients dropped. Finally, 10 babies were included in the br and VD groups (VD-br group: four males and six females), 17 babies were included in the fo and VD groups (VD-fo group: 10 males and seven females), eight babies were included in the br and CS groups (CS-br group: four males and four females), and 27 babies were included in the fo and CS groups (CS-fo group: 15 males and 12 female). In total, 59 stool samples at 40 days of age (40 days), 60 samples at 3 months of age, and 53 samples at 6 months of age were collected.

A total of 62 infants were enrolled (i.e., 33 males; 29 female; male/female = 1.14), with a mean gestational age of 39 w 1 d ± 1 w 1 d (w: week; d: day; range: 37–42 weeks), birth weight of 3,268 ± 393 g (2,500–4,400 g), 25th and 75th percentile (P25/P75) birth lengths of 49.0/50.8 cm (45.0–55.0 cm), and birth head circumference of 33.0/34.0 cm (32.0–35.0 cm).

Some babies were exposed to antibiotic therapy, including one baby (with maternal postnatal oral cephalosporin administration) in the VD-br group, two babies (one baby with maternal postnatal antibiotics and one with infantile antibiotics administration) in the VD-fo group, and 10 babies (two babies with maternal prenatal antibiotics, three babies with maternal postnatal antibiotics, four babies with infantile antibiotics, and one baby with both maternal postnatal antibiotics and infantile antibiotics administration) in the CS-fo group. Oral vitamin D was supplied to all babies except for three babies in the VD-fo group and six babies in the CS-fo group. These babies were fed breast milk or specific formulas from birth to 6 months of age without any other probiotics or prebiotics. Some babies had eczema, food allergies, and infections of the respiratory or intestinal system. However, none of them suffered from other chronic diseases or severe infections or were hospitalized during follow-up.

The basic characteristics showed no significant differences among the four groups (Table 1).

**Table 1:** Basic characteristics.

**Group**	**VD-br**	**VD-fo**	**CS-br**	**CS-fo**	***p*** **value**
Total number (n)	10	17	8	27	—
Male/female	4/6	10/7	4/4	15/12	0.832^b^
Usage of antibiotics(yes/no)	1/9	2/15	0/8	10/17	0.064^b^
Gestational age (w: week. d: day)	39 w 4 d ± 6 d	39 w 3 d ± 6 d	38 w 6 d ± 1w	38 w 5 d ± 1 w 3 d	0.185^c^
Birth weight (g)	3,233 ± 343	3,241 ± 344	3,320 ± 435	3,285 ± 445	0.959^c^
Birth length(cm)	50.0 ± 1.4	49.8 ± 1.9	45.0	50.0/51.5^a^	0.335^c^
Head circumference (cm)	32.5/34.0^a^	33.6 ± 1.1	—	33.7 ± 0.5	0.775^c^
Weight (g) in 40 d	4,650/4,963^a^	4,655 ± 511	4,500/4,700^a^	4,698 ± 67	0.752^c^
Length (cm) in 40 d	55.6 ± 1.3	55.8 ± 1.6	53.5/55.0^a^	54.5/56.4^a^	0.196^c^
Head circumference (cm) in 40 d	36.4/38.1^a^	37.2/38.3^a^	36.5/38.5^a^	37.0/38.6^a^	0.964^c^
Weight (g) in 3 m	6,250/6950^a^	6,604 ± 584	5,800/6,400^a^	6,200/7,000^a^	0.217^c^
Length (cm) in 3 m	60.0/63.0^a^	61.3/62.9^a^	57.0/62.0^a^	60.0/63.7^a^	0.174^c^
Head circumference (cm) in 3 m	39.8/41.1^a^	39.8/41.1^a^	38.5/40.0^a^	40.0/42.0^a^	0.112^c^
Weight (g) in 6 m	8,400 ± 860	8,055/8850^a^	8,294 ± 1,026	7,900/8,700^a^	0.928^c^
Length (cm) in 6 m	65.5/68.8^a^	66.5/70.0^a^	65.0/68.0^a^	459.5 ± 2.7	0.560^c^
Head circumference (cm) in 6 m	41.1/44.3^a^	43.0/45.0^a^	41.0/43.0^a^	43.0/44.4^a^	0.091^c^
Eczema or food allergies before 2 years old (yes/no)	8/2	9/8	3/5	15/12	0.330^b^
Times of respiratory or intestinal infections before 2 years old	3/5	5 ± 3	4 ± 3	4 ± 2	0.987^c^

a *.The values of the 25th and 75th percentile (P25/P75)*.

b *.Intergroup differences were evaluated using the χ2 test for categorical variables*.

c *.Variables were statistically tested using the Kruskal-Wallis test*.

### α Diversity

**Figure 1:**
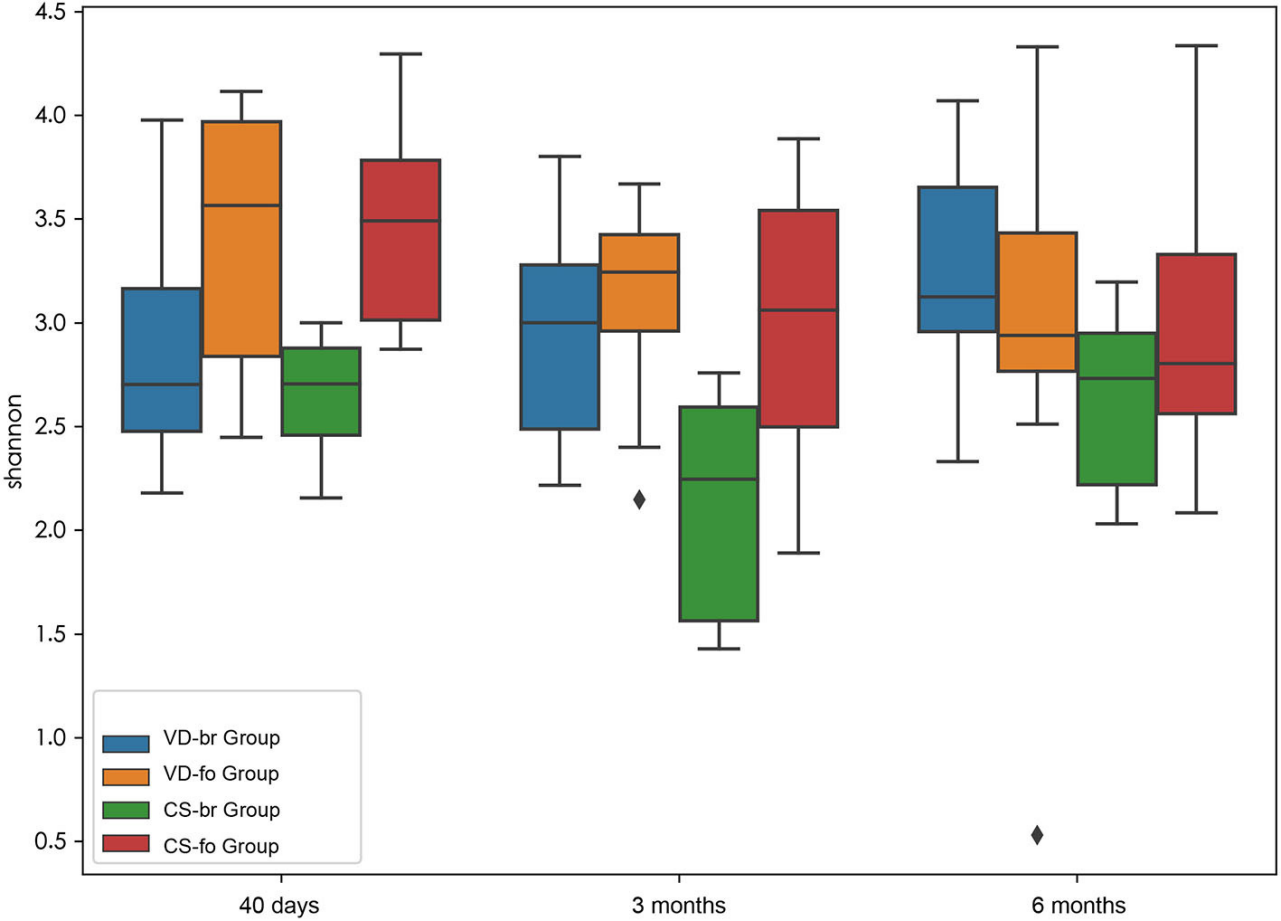
α diversity measurements using Shannon index values indicated gut microbiota diversity. Y-axis represents the values of Shannon index. Boxplots compare α diversity of gut microbiota in 40 days (40 d), 3 months (3 m), and 6 months (6 m) of age among different groups. Boxes show 25th to 75th percentiles and the median line, and whiskers indicate minimum to maximum values. Statistical significance was evaluated by Wilcoxon test within each group and a two-tailed *t* test among different groups, using *p* ≤ 0.05 as the measure of significance.

α diversity (within-sample diversity) measurements using Shannon index values indicated the gut microbiota diversity (Figure 1).

From 40 days to 6 months of age, the α diversity (reported as the Shannon index) indicated that the bacterial communities did not change significantly in the VD-br, VD-fo, and CS-br groups but decreased significantly at 3 months and 6 months of age in the CS-fo group (40 days vs. 3 months: Shannon index 3.48 ± 0.45 vs. 3.01 ± 0.58, *p* = 0.0005; 40 days vs. 6 months: 3.48 ± 0.45 vs. 2.94 ± 0.54, *p* = 0.0005; according to the Wilcoxon test).

At 40 days of age, the Shannon index showed a lower count in the br infants than in the fo infants, regardless of the delivery mode (VD-br vs. VD-fo: Shannon index = 2.91 ± 0.65 vs. 3.42 ± 0.57, *p* = 0.05; VD-br vs. CS-fo: 2.91 ± 0.65 vs. 3.48 ± 0.45, *p* = 0.006; CS-br vs. VD-fo: 2.65 ± 0.30 vs. 3.42 ± 0.57, *p* = 0.003; CS-br vs. CS-fo: 2.65 ± 0.30 vs. 3.48 ± 0.45, *p* = 6.7E-05; according to a two-tailed *t*-test). At 3 months of age, the α diversity was significantly lower in the CS-br group than in the other three groups (CS-br vs. VD-br, VD-fo, and CS-fo: 2.12 ± 0.54 vs. 2.98 ± 0.54, 3.13 ± 0.43, and 3.01 ± 0.58, respectively, *p* = 0.005, 4.0E-05, and 0.0006, respectively; according to a two-tailed *t*-test) but showed no significant differences at 6 months of age.

### β Diversity

**Figure 2:**
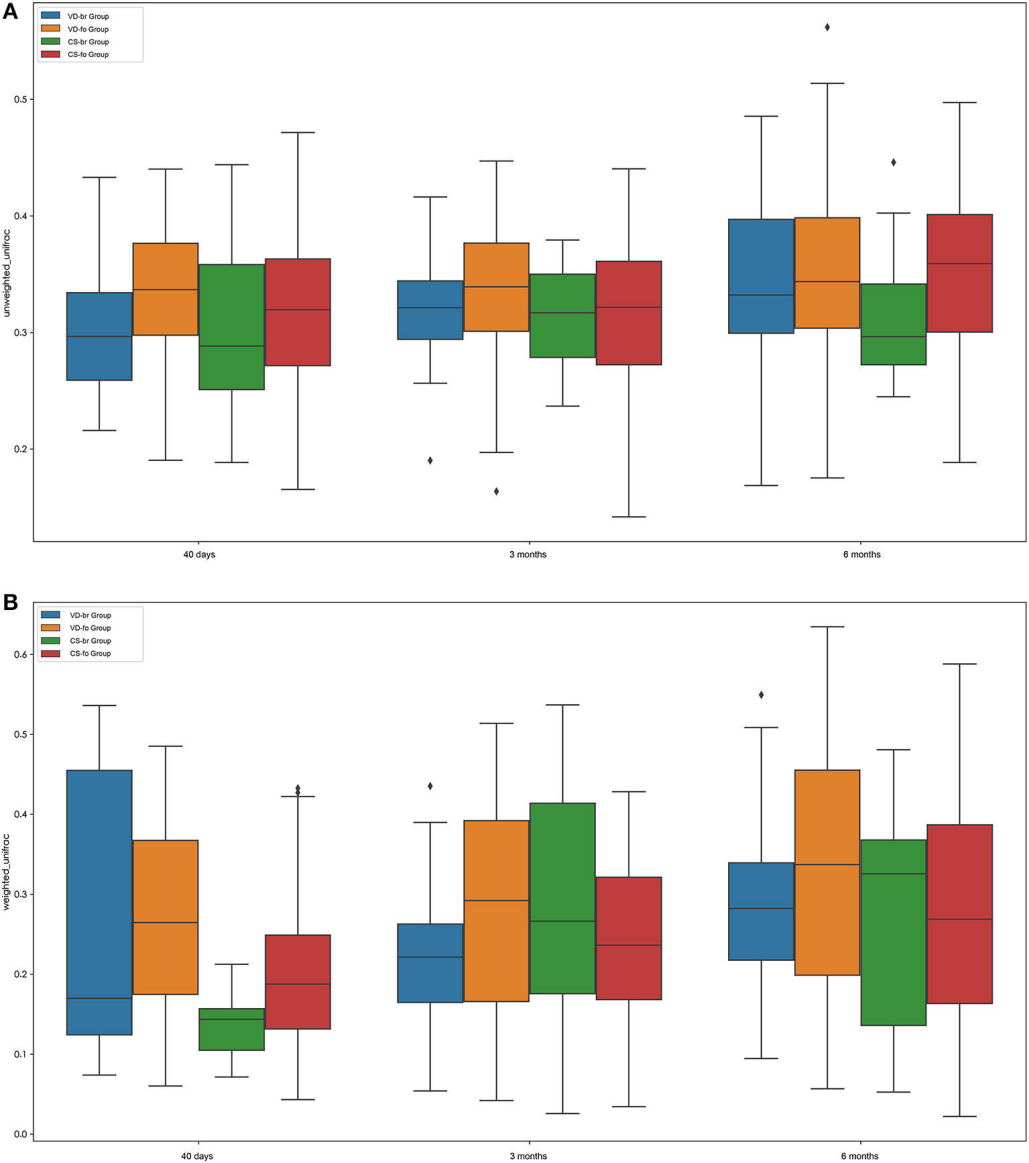
β diversity measurements by Unweighted UniFrac **(A)** and Weighted UniFrac **(B)**. Boxplots compare β diversity of gut microbiota in 40 days (40 d), 3 months (3 m), and 6 months (6 m) of age among different groups. Boxes show 25th−75th percentiles and the median line, and whiskers indicate minimum to maximum values. Statistical significance was evaluated by a two-tailed *t* test, using *p* ≤ 0.05 as the measure of significance.

The β diversity (between-sample diversity) was measured according to the unweighted UniFrac and weighted UniFrac values (Figure 2; according to a two-tailed *t*-test).

The β diversity of the infant gut microbial community increased steadily during the first 6 months of age in all groups, although the difference was not significant between 3 months and 40 days of age in the VD groups, including the VD-br and VD-fo groups.

At 40 days of age, the β diversity was higher in the VD groups than in the CS groups, regardless of the feeding type (VD-br vs. CS-br, *p* = 0.0008; VD-br vs. CS-fo, *p* = 6.0720E-06; VD-fo vs. CS-br, *p* = 8.0453E-08; VD-fo vs. CS-fo, *p* = 9.9344E-16). In the VD groups, there was no significant difference between the br infants and fo infants. However, in the CS groups, the fo infants showed higher β diversity than the br infants (CS-fo vs. CS-br, *p* = 0.0014). At 3 months of age, the β diversity in the VD-br and CS-fo groups was much lower than that in the VD-fo (VD-br vs. VD-fo, *p* = 0.0095; CS-fo vs. VD-fo, *p* = 0.0002) and CS-br groups (VD-br vs. CS-br, *p* = 0.0441; CS-fo vs. CS-br, *p* = 0.0269), while at 6 months of age, the β diversity was the highest in the VD-fo group (VD-fo vs. VD-br, CS-br, and CS-fo, *p* = 0.0422, 0.0414, and 0.0003, respectively) according to the weighted UniFrac value.

### Fecal Microbial Composition

**Figure 3:**
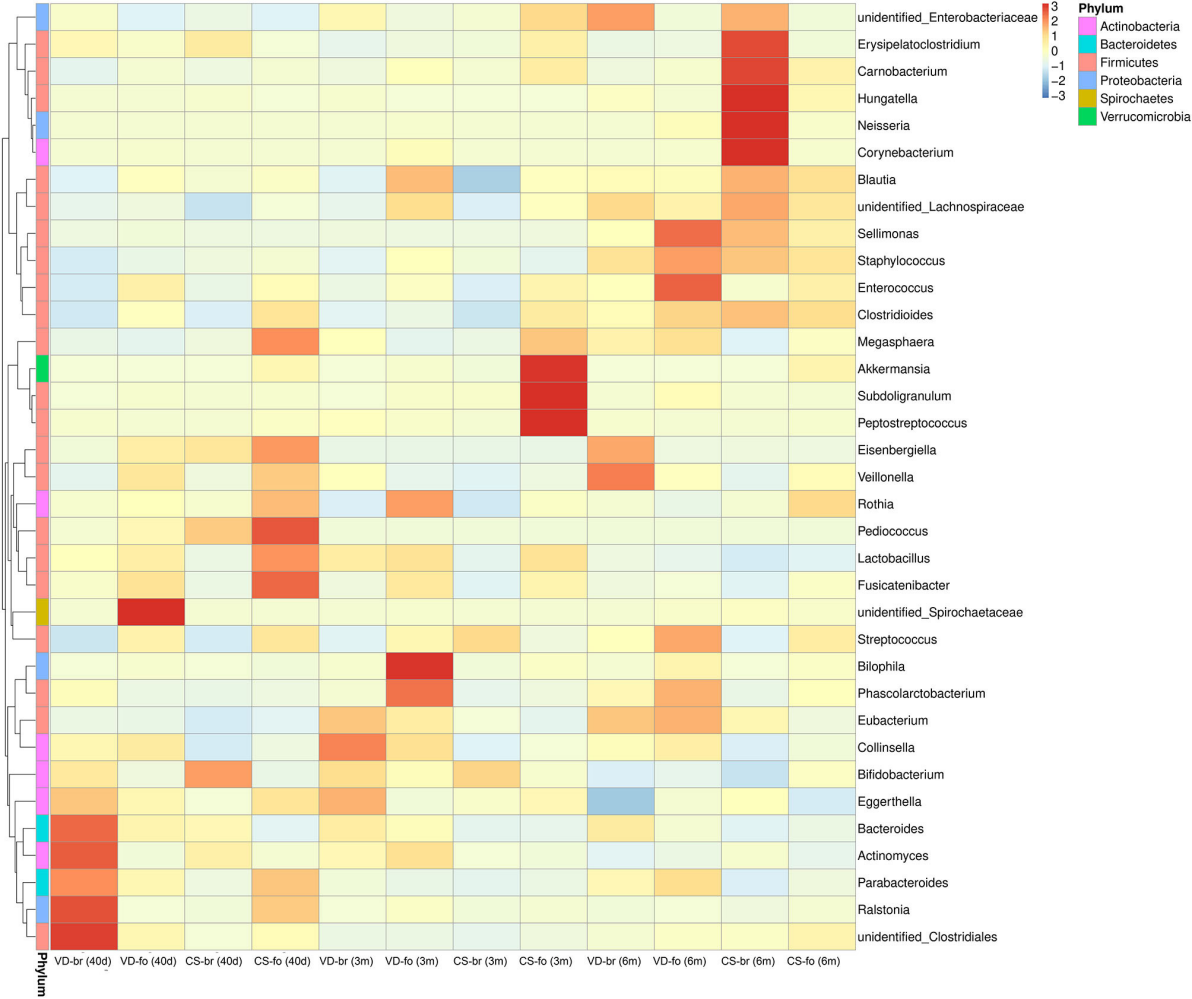
The OTUs heatmap at genus level. The relative abundance of OTUs was assessed across all samples, and OTUs were clustered in a heatmap according to their co-occurrence at genus level. Clustering was performed as a type of hierarchical clustering method to interpret the distance matrix using average linkage. The dendrogram provides the genus designation along the right Y-axis and the abundance relationship across all samples for each genus along the left Y-axis. The color scale for the heatmap is shown in the upper right corner of the figure.

**Figure 4:**
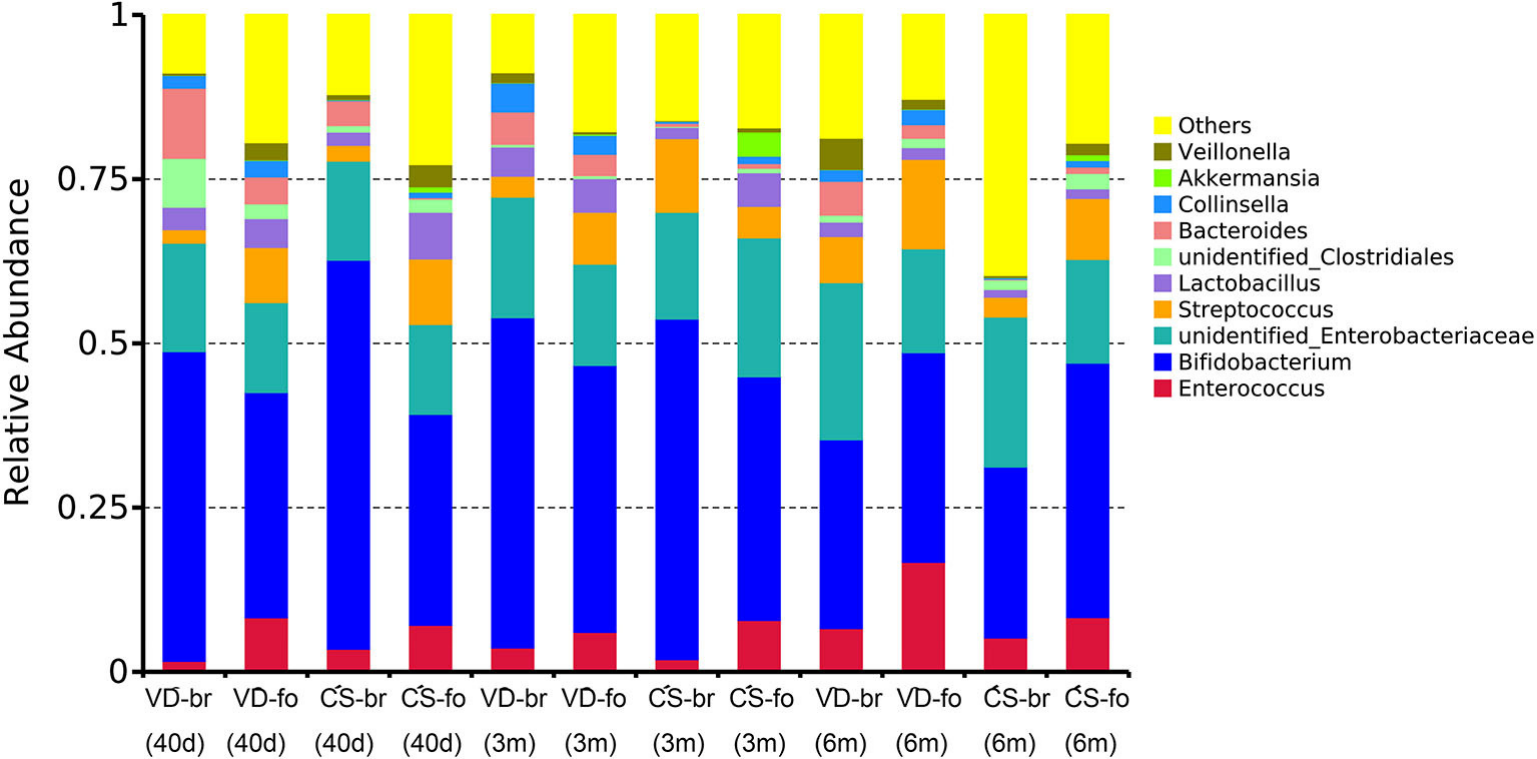
The 10 most abundant bacteria of gut microbiota at genus level. Relative abundance estimates of the 10 most dominant bacteria at genus level in 40 days, 3 months, and 6 months of age among different groups.

The relative abundance of operational taxonomic units (OTUs) was assessed across all samples, and the OTUs were clustered in a heatmap according to their co-occurrence at the genus level (Figure 3). The 10 most abundant bacteria of the gut microbiota at the genus level are shown in Figure 4.

*Bifidobacterium* was the most predominant genus, and *Enterobacteriaceae* was the second most predominant genus in all groups at all time points. *Bifidobacterium* accounted for 47.1, 50.3, and 28.7% in the VD-br group, 34.3, 40.6, and 31.9% in the VD-fo group, 59.2, 51.8, and 26.0% in the CS-br group, and 32.1, 37.1, and 38.7% in the CS-fo group at 40 days, 3 months, and 6 months of age, respectively. *Enterobacteriaceae* accounted for 16.5, 18.3, and 23.9% in the VD-br group, 13.7, 15.4, and 15.8% in the VD-fo group, 15.1, 16.2, and 22.8% in the CS-br group, and 13.7, 21.1, and 15.8% in the CS-fo group at 40 days, 3 months, and 6 months of age, respectively.

At 40 days of age, *Bacteroides* ranked third in the VD groups (10.7% in the VD-br group and 4.1% in the VD-fo group) and CS-br group (3.8%), followed by *unidentified_Clostridiales* and *Lactobacillus* in the VD-br group and *Streptococcus* and *Enterococcus* in the VD-fo and CS-br groups. However, in the CS-fo group, the third and fourth most abundant were *Streptococcus* (10.0%) and *Enterococcus* (7.2%), respectively, with *Bacteroides* being the least abundant (0.2%).

At 3 and 6 months of age, *Streptococcus* (4.8–13.6%) and *Enterococcus* (1.9–16.8%) ranked third or fourth in all groups, except for the VD-br group at 3 months of age, in which the third was still *Bacteroides* (4.9%), followed by *Lactobacillus* (4.4%).

### Comparison of Gut Microbiota in Different Groups

In our study, solid foods were introduced from 4 to 6 months of age; therefore, these foods only affected the last time point at 6 months of age.

**Figure 5:**
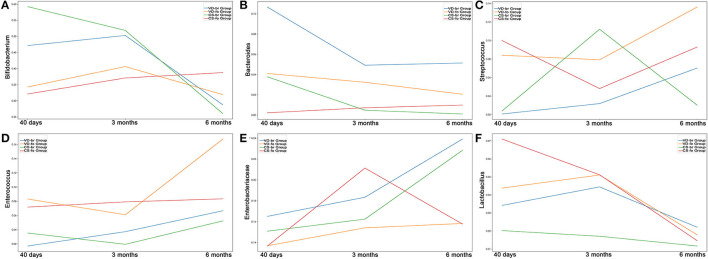
The relative abundance curves of **(A)** *Bifidobacterium*, **(B)** *Bacteroides*, **(C)** *Streptococcus*, **(D)** *Enterococcus*, **(E)** *Enterobacteriaceae* and **(F)** *Lactobacillus* in different groups from 40 days to 6 months old. Y-axis represents the median line of relative abundance. **(A)** In CS-br group, *Bifidobacterium* decreased significantly in 6 months of age, compared with that in 40 days (*p* = 0.0313) and 3 months of age (*p* = 0.0469). In 40 days of age, there were significant differences between VD-br and CS-fo group (*p* = 0.0294), CS-br and CS-fo group (*p* = 6.789E-05), and CS-br and VD-fo group (*p* = 0.0057). **(B)** In VD-br group, *Bacteroides* decreased significantly in 3 months of age, compared with that in 40 days (*p* = 0.0078). In VD-fo group, Bacteroides decreased significantly in 6 months of age, compared with that in 3 months (*p* = 0.0353). In 40 days of age, there were significant differences between CS-fo group and other groups (vs. VD-br *p* = 0.0003; vs. VD-fo *p* = 0.0189; vs. CS-br *p* = 0.0004). In 3 months and 6 months of age, *Bacteroides* in VD-br group was different from CS-fo group (3m *p* = 0.0179; 6m *p* = 0.0240). **(C)** In VD-br group, *Streptococcus* increased significantly in 6 months of age, compared with that in 40 days (*p* = 0.0234) and 3 months (*p* = 0.0234). In CS-fo group, Streptococcus decreased significantly in 3 months of age (*p* = 0.0074) and then increased in 6 months of age (*p* = 0.0415). In 40 days of age, there were significant differences between VD-br and CS-fo group (*p* = 0.0065), and CS-br and CS-fo group (*p* = 0.0197). **(D)** *Enterococcus* was not significantly changed over time. In 40 days of age, there were significant differences between VD-br and CS-fo group (*p* = 0.0002), and CS-br and CS-fo group (*p* = 0.0403). In 3 months of age, there were significant differences between VD-fo and CS-br group (*p* = 0.0312), and CS-fo and CS-br group (*p* = 0.0268). **(E)** *Enterobacteriaceae* decreased significantly in 6 months of age in CS-fo group, compared with that in 3 months (*p* = 0.0083). **(F)** In VD-fo group, *Lactobacillus* decreased significantly from 3 months to 6 months of age (*p* = 0.0295). In CS-fo group, *Lactobacillus* decreased significantly from 40 days to 6 months of age (*p* = 0.0003). The differences of *Lactobacillus* among different groups were not significant.

**Figure 6:**
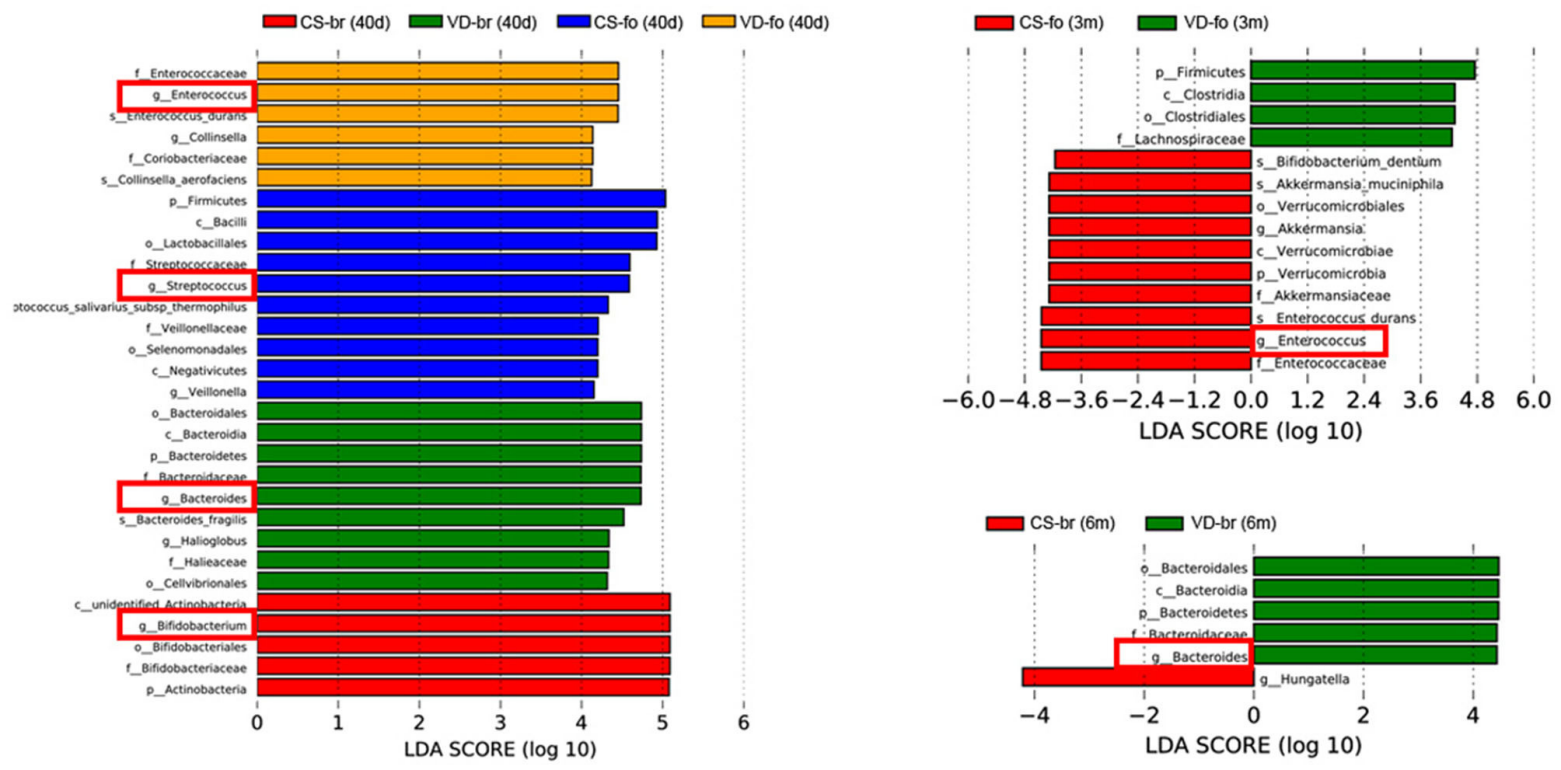
LDA scores for the bacterial taxa differentially abundant among different groups (LDA > 4.0). Bars with different colors indicate taxa were enrichment in corresponding groups. Red squares mark the main different microbiota at genus level.

The curves and comparison of *Bifidobacterium, Bacteroides, Streptococcus, Enterococcus, Enterobacteriaceae*, and *Lactobacillus* in different groups are shown in Figures 5, 6.

At 40 days of age, the abundance of *Bifidobacterium* was much higher, while the abundance of *Streptococcus* and *Enterococcus* was much lower in br infants than in the CS-fo group infants. However, *Bifidobacterium* was higher in infants delivered by CS who were fed breast milk than those who were fed formula, including VD-fo subjects. The lowest *Bacteroides* abundance was observed in the CS-fo group compared with the other groups.

At 3 months of age, *Enterococcus* was higher in the fo infants than in the CS-br group. However, for the infants delivered by VD, there was no difference between the fo and br babies.

The abundance of *Bacteroides* in the VD-br group was still higher than that in the CS-fo group at 6 months of age. However, no difference was shown between the other groups.

## Discussion

The results showed that α diversity was associated with the feeding type and delivery mode. Studies have shown that infants who are fed breast milk have lower microbial diversity than those who are fed formula and whose gut microbiota are more diverse (Roger and McCartney, 2010; Roger et al., 2010; Bridgman et al., 2017). The difference in gut microbial diversity between br and fo babies has also been reported in animal research (He et al., 2018). We also found that at 40 days of age, the α diversity was lower in the br infants than in the fo infants, regardless of the delivery mode. At 3 months of age, the α diversity was significantly lower in the CS-br group than in the other 3 groups but showed no significant differences at 6 months of age after solid food introduction. A previous study indicated that the α diversity was increased and the bacterial community was more complex in fo infants than in that of br infants at newborn and after 4 months (Bäckhed et al., 2015), which was consistent with our study. The study showed a higher α diversity in CS-delivered infants than in those delivered by the vaginal route (Bäckhed et al., 2015); this finding was inconsistent with that of our study, which was probably because we considered the interactions of feeding types and delivery modes to avoid confounding factors. In other words, breastfeeding might neutralize the influence of CS on gut microbiota, thereby leading to a lower diversity, which is similar to the findings in our study. In infants, the predominance of infant-type *Bifidobacteria* during breastfeeding results in low bacterial diversity, although it is beneficial for babies' health.

The literature indicates that *Bifidobacterium* is present in the first few months and decreases with age to almost zero by 18 months old (Yassour et al., 2016). We found that in the CS-br group, *Bifidobacterium* decreased significantly over time at 6 months of age. In the other groups, *Bifidobacterium* also decreased, although the differences were not significant. *Enterobacteriaceae* also decreases with time (Pannaraj et al., 2017; Baumann-Dudenhoeffer et al., 2018). *Enterobacteriaceae* decreased significantly at 6 months of age in the CS-fo group compared with that at 3 months. In the other groups, *Enterobacteriaceae* increased over time, although the differences were not significant.

Our study showed that breastfeeding is associated with higher levels of *Bifidobacterium*, which is in accordance with other studies (Levin et al., 2016; Liu et al., 2016; Stewart et al., 2018). *Bifidobacterium* possesses multiple benefits that are reported to be associated with a diminished risk of allergic diseases (Björkstén et al., 2001) and excessive weight gain (Dogra et al., 2015). The growth parameters of babies before 6 months old and the incidence of allergic diseases and infections before 2 years of age were not significantly different among the different groups in our study. Perhaps, a longer follow-up time is required to determine the long-term influences of different feeding types and delivery modes on infants. In addition to feeding types, delivery modes also have impacts on infant gut *Bifidobacterium*. The gut microbiota in infants born by VD shares characteristics with that of the maternal vagina and intestinal tract, whereas in infants born by CS, the gut microbiota is similar to that of the maternal skin but lacks bacteria from the vaginal community in the early life period. The gut microbiota of VD newborns was enriched in *Bifidobacterium*. Mother-newborn transmission of *Bifidobacterium* was also observed in infants delivered by CS, although the frequency was lower compared with infants delivered vaginally (Bäckhed et al., 2015). We found that at 40 days of age, the abundance of *Bifidobacterium* was much higher in the CS-br group than in the CS-fo group but did not differ between the VD-br and VD-fo groups. This finding indicated the impact of the delivery mode on *Bifidobacterium* in the early period of life. VD might neutralize the influence of feeding type on *Bifidobacterium*. The differences in *Bifidobacterium* disappeared at 3 and 6 months of age among the different groups.

Studies have indicated that breast milk maintains a lower abundance of *Enterococcaceae* and *Streptococcaceae* in the gut (Roger and McCartney, 2010; Bäckhed et al., 2015; Laursen et al., 2016), which is consistent with our results. Previous research has investigated the microbiota of the mothers' skin and vagina 1 h before delivery and the neonates' skin, oral mucosa, nasopharyngeal aspirate, and meconium within a short time after delivery, and a greater abundance of *Streptococcus* was observed in the samples of babies born *via* CS and samples of the mothers' skin than in the samples of babies born vaginally and samples of the mothers' vagina (Dominguez-Bello et al., 2010). We found that at 40 days of age, the abundance of *Streptococcus* and *Enterococcus* in br infants (i.e., VD-br and CS-br groups) was significantly lower than that in the CS-fo group but nonsignificantly lower than that in the VD-fo group. At 3 months of age, *Enterococcus* was still significantly lower in br infants who were born by CS than in fo infants. However, for infants delivered by VD, the difference was not significant between fo and br babies. These results indicated that in addition to the food type, the VD delivery mode might have a large impact on the abundance of *Streptococcus* and *Enterococcus*, thereby neutralizing the impacts caused by different feeding types. Moreover, researchers have indicated that higher levels of *Streptococcus sp*. occur in patients suffering from type 1 diabetes (Levin et al., 2016). Although these bacteria may have additional negative effects, limited information is available.

## Conclusion

Differences in gut microbiota among infants based on specific interactions of feeding type and delivery mode were obtained in this study, which included a larger cohort than in previous studies. Moreover, babies were exclusively fed breast milk or a single brand of formula in our study rather than mixed food types. It is worth mentioning that breastfeeding may neutralize the effects of CS on the microbial α diversity, and VD may neutralize the impacts of formula on the prevalence of specific genera, such as *Bifidobacterium, Streptococcus*, and *Enterococcus*, which should not be ignored in future research. Breastfeeding and vaginal delivery (VD) are natural processes that confer many benefits to infants' gut microbiota and health. We can conclude from our research that breastfeeding and VD can decrease the probable adverse impacts caused by formula and CS on gut microbiota.

## Data Availability Statement

The datasets presented in this study can be found in online repositories. The names of the repository/repositories and accession number(s) can be found below: https://www.ncbi.nlm.nih.gov/, sra/PRJNA633365.

## Ethics Statement

The studies involving human participants were reviewed and approved by Ethics Institutional Review Board of Peking Union Medical College Hospital. Written informed consent to participate in this study was provided by the participants' legal guardian/next of kin.

## Author Contributions

ZL initiated and supervised this research. WZ, YZ, NZ, JM, DW, CZ, HM, and HW performed the experiments. JM and ZL analyzed the data, wrote the manuscript, and prepared the figures. All authors contributed to the article and approved the submitted version.

## Conflict of Interest

The authors declare that the research was conducted in the absence of any commercial or financial relationships that could be construed as a potential conflict of interest.

## Publisher's Note

All claims expressed in this article are solely those of the authors and do not necessarily represent those of their affiliated organizations, or those of the publisher, the editors and the reviewers. Any product that may be evaluated in this article, or claim that may be made by its manufacturer, is not guaranteed or endorsed by the publisher.
